# Sample Preparation for MALDI-TOF Mass Spectrometry of Model Prebiotic Reactions in Simulated Ocean World Environments

**DOI:** 10.1021/acsomega.5c07633

**Published:** 2025-10-20

**Authors:** Katherine A. Dzurilla, Elin C. Herndon, Laura M. Barge, Jay G. Forsythe

**Affiliations:** † 53411 NASA Jet Propulsion Laboratory, California Institute of Technology, Pasadena, California 91109, United States; ‡ Department of Chemistry and Biochemistry, 2343 College of Charleston, Charleston, South Carolina 29424, United States

## Abstract

The prebiotic potential of ocean worlds such as Europa and Enceladus, while not well constrained, is vital in the search for potential life elsewhere. To better understand this potential, laboratory studies provide experimental insight into the chemical environment(s) of ocean worlds and any constraints to hold prebiotically relevant systems. However, simulated ocean world solutions present a technical challenge for analytical techniques such as mass spectrometry (MS), as the high saline content required for simulating ocean world environments results in ion suppression and reduced sensitivity. Here, we compare three sample preparation techniques, C4 ZipTips, C18 ZipTips, and on-plate washing, for matrix-assisted laser desorption/ionization – time-of-flight (MALDI-TOF) MS of saline solutions similar to environments expected in ocean worlds. We determined that using C18 ZipTips was optimal when paired with 2,5-dihydroxybenzoic acid (DHB) matrix, whereas on-plate desalting was optimal when paired with α-cyano-4-hydroxycinnamic acid (CHCA) matrix. The methodology described here presents simple, low-volume sample preparation strategies for MALDI-TOF MS of model prebiotic reactions in saline solutions.

## Graphical abstract

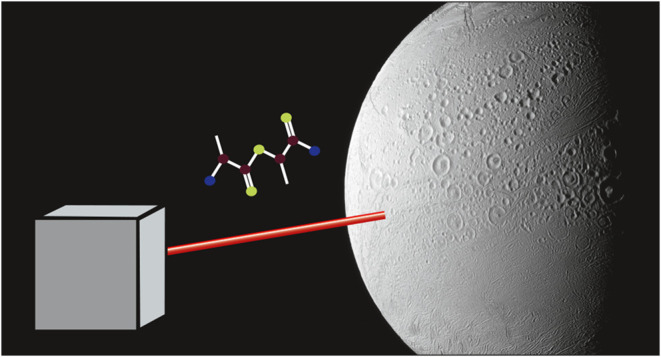

## Introduction

Ocean worlds in our solar system, such as Europa and Enceladus, are important targets in the search for life. These planetary bodies differ from traditional rocky planets in that they contain an icy crust and a subsurface ocean in between the ice layer and a rocky core (Figure 1).^1–3^ While the specific composition, depth, and accessibility of the subsurface ocean environments will be unique to each planetary body, their aqueous environments and water/rock interactions make them an important location when looking for prebiotic or biological systems. Ocean worlds are projected to contain features thought to be conducive for life to arise based on our understanding of the conditions on early Earth that led to life’s emergence.

**Figure 1:**
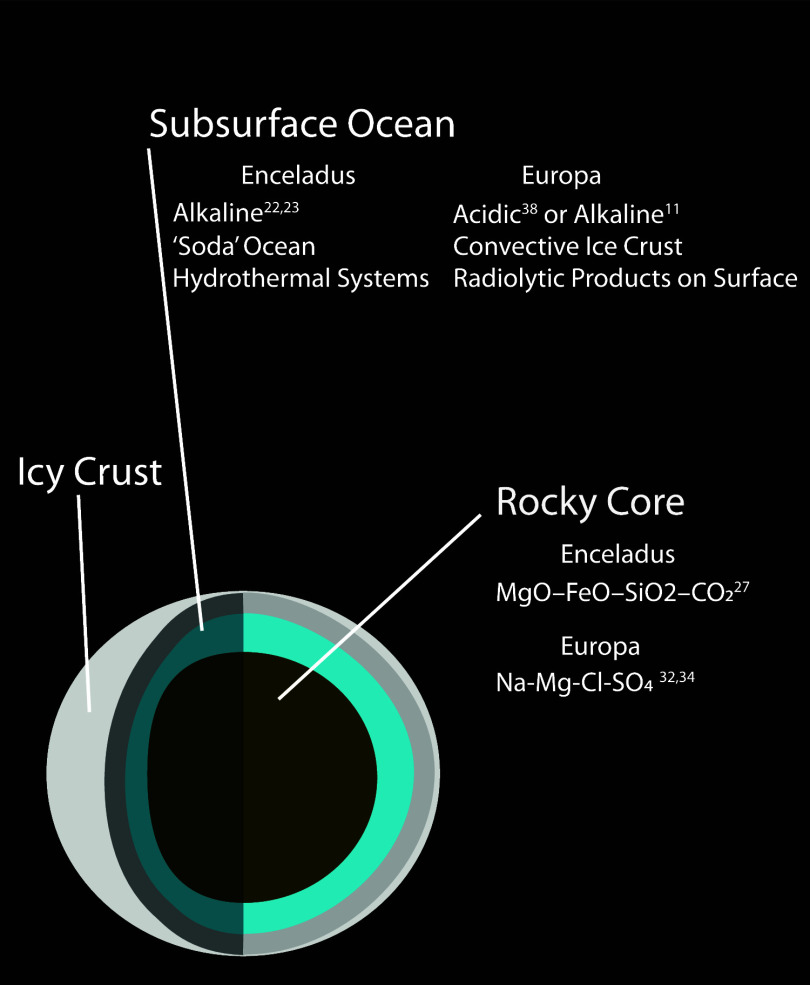
Diagram of the ocean world and characteristics unique to Europa and Enceladus.

Carbon, hydrogen, oxygen, nitrogen, phosphorus, and sulfur (CHONPS) are essential elements required for biochemistry as we know it. Organics, including aromatics with nitrogen and oxygen, have been detected on Enceladus through data obtained from the Cassini mission.^4,5^ Phosphorus and potentially sulfur have also been detected on Enceladus,^6,7^ with the specific phosphorus species observed in biological activity (phosphate) being the predominant phosphorus form. Additionally, Enceladus is thought to house hydrothermal systems;^8^ such environments may produce prebiotic reactions and have been proposed to be locations for the origins of life on Earth.^9^ On Europa, CHONPS elements have also been detected on the surface, with CO_2_ and O_2_ observed as products from radiolytic processes occurring on the surface, along with ammonia and sulfate salts thought to be sourced from the subsurface ocean.^10–12^ Convection within the ice crust of Europa could deliver these elements into the ocean environment where further reactions of potential biological importance could be occurring.^13^ While phosphorus has not been detected to date, the Europa Clipper^14^ mission may provide more information on phosphorus presence on Europa. Hydrothermal systems are also thought to be possible within the subsurface ocean of Europa;^15^ however, no direct evidence has been obtained. These ocean worlds therefore contain many of the elemental building blocks required for prebiotic chemistry, particularly if CHONPS organics are present with the hydrothermal systems. Ocean worlds have increasingly become a target for future NASA missions, as the Enceladus Orbilander was recommended by the most recent NASEM decadal survey.^16^

Investigating habitability constraints within these ocean world environments is important in the search for life, particularly in the ability of the subsurface oceans of Europa and Enceladus to perform prebiotic reactions. Compounds relevant to prebiotic and biological systems, such as amino acids, are thought to be possible on ocean worlds as they have been discovered in asteroids potentially similar to the core of Enceladus^17^ and have been found to create dimers under Enceladus-like conditions.^18^ Polymers thought to be potentially prebiotic might indicate an evolving system capable of hosting or evolving life, or that complex life might be present (however, this would be heavily dependent on the environmental context).^19^ For example, depsipeptides, oligomers of amino acids and hydroxy acids, are thought to be potential precursors to modern-day peptides as activated ester linkages between hydroxy acids enable amide bond formation.^20,21^ Investigating the potential for ocean worlds to produce organic oligomeric compounds, such as peptides and depsipeptides, through laboratory studies can provide information on their habitability and the potential prebiotic state of their environments.^19^ However, these subsurface ocean environments are expected to contain concentrated salts, such as NaCl and MgCl_2_.^22–25^ Experimental studies investigating the prebiotic potential of these ocean worlds, therefore, would incorporate high saline conditions to accurately represent ocean world environments.

### Ocean World Geochemistry

Europa and Enceladus both maintain an ice crust and salty ocean; however, the more specific characteristics of their subsurface oceans will be dependent on their unique geochemical environments and the equilibrium of their water/rock interactions. As previously stated, the Enceladus ocean is expected to contain high concentrations of salts (Table 1), such as NaCl and Na_2_CO_3_ (0.05–0.2 mol/kg and 0.02–0.1 mol/kg respectively),^22–24^ and organics^4,5^ based on Cassini mission data obtained through samples taken from Enceladus southern plume material. An alkaline environment (pH ∼ 9)^22^ is expected along with hydrothermal and serpentinizing systems,^8,26^ where fluid moving within the hydrothermal systems would circulate through a porous layer in Enceladus core.^27,28^ Quartz, talc, and carbonate minerals in a MgO–FeO–SiO_2_–CO_2_ system on the sea floor would provide long-term buffering of the ocean environment.^27^

**Table 1:** Plausible Conditions of Enceladus and Europa Based on the Current Literature

	Enceladus	Europa
pH	∼9.5 ^22,23^	acidic^38^ or alkaline^11^
NaCl	0.05–0.2 mol/kg	0.1–1.6 M Na^+^ ^25,35,36^
		0.06–0.3 M Cl^–^ ^25,35,36^
Na_2_CO_3_	0.02–0.1 mol/kg	
Mg_2_Cl		0–2.9 M Mg^2+^ ^25,35,36^
		0.06–0.3 M Cl^–^ ^25,35,36^
MgSO_4_		0–2.9 M Mg^2+^ ^25,35,36^
		0–3.6 M SO_4_^2–^ ^25,35,36^

Less is known about Europa’s expected subsurface ocean^3,29^ and its geochemical environment (Table 1). Spectral data from the surface and geochemical modeling can provide some insight into the ocean environment, as materials exposed on the surface are thought to be sourced from the ocean due to the convective processes of Europa’s ice crust.^30,31^ Radiolytic and freezing processes are expected to alter these ocean relics, as they move through the crust and are exposed to the surface. For example, the MgSO_4_ detected on Europa’s surface during the Galileo mission,^10,12^ while originating from the ocean, could be produced from Mg^+^ and Cl^–^ ions as they are exposed to the freezing and irradiation environments containing sulfur radicals from Io.^32,33^ Models based on these observations predict a subsurface ocean containing a Na–Mg–Cl-SO_4_ ^32,34^ system producing a range of ions such as Na^+^ (0.1–1.6 M), Mg^2+^(0–2.9 M), Cl^–^ (0.06–0.3 M), and SO_4_^2–^ (0–3.6 M)^25,35,36^ that would react to form various salts such as MgCl_2_, NaCl, and MgSO_4_.^11,34^ The pH of Europa’s ocean is debated, as it will be greatly influenced by the convective ice processes in the crust, the geological makeup of the core, and the equilibrium of the water/rock interactions.^13^ Radiolytic products of O_2_ and H_2_O_2_ oxidants are expected to be produced on the surface of Europa, on the order of 10^9^ mol/year,^37^ and while the rate of oxidant delivery to the subsurface ocean is debated,^13,37^ delivery could be accomplished as quickly as ∼ 1 Myr,^13^ leading to an acidification of the subsurface through interactions with sulfide (H_2_S).^38^ Alkaline conditions in the Europa ocean could be maintained with hydrothermal buffering;^11^ however, hydrothermal activity has not been confirmed.

### Analytical Challenges in High Saline Environments

Laboratory studies investigating prebiotic reactions within highly saline environments relevant to Europa and Enceladus present technical challenges for sample analysis. For peptides and depsipeptides, a range of analytical techniques has been used, such as mass spectrometry (MS),^39^ liquid chromatography–mass spectrometry (LC-MS),^21,40^ and Fourier transform infrared spectroscopy (FTIR) spectroscopy.^41^ Additionally, other techniques, such as high-performance liquid chromatography coupled with UV–vis detection (HPLC-UV), can be used. However, high salt content can lead to source scattering in UV–vis measurements, and depsipeptides/peptides with residues lacking in chromophores (e.g., aromatic side chains) have low sensitivity when detected solely by UV–vis.

MS techniques are ideal for the analysis of peptides and depsipeptides, yet they are also affected by high salt (nonvolatile) concentrations. Salts within MS samples increase adduct formation and suppress analyte ionization, decreasing overall S/N.^42–44^ For example, concentrations as low as >0.5 mM NaCl were found to increase adducted features in electrospray (ESI) mass spectra, decreasing analyte signal strength in studies with modern biological proteins.^45^

Matrix-assisted laser desorption/ionization–time-of-flight (MALDI-TOF) MS is an analytical technique that irradiates the analyte and the laser-absorbing matrix to produce gas-phase ions. Sample preparation (matrix choice, sample deposition method, etc.) is critical and can greatly influence MALDI spectral quality.^46^ MALDI is slightly more tolerant of nonvolatile salts than ESI; nevertheless, high saline content decreases analyte S/N and increases adduct formation, reducing sensitivity.^47^ Desalting is relatively common for MALDI-TOF MS analysis of biological samples. For example, on-plate rinsing with a matrix was shown to increase spectral quality from biological samples from seawater environments.^48^ ZipTips, single-use micropipette tips that include a chromatography resin, can also be used to desalt samples before MS analysis.^49^

Here, we performed a set of experiments to optimize MALDI sample preparation for model prebiotic oligomer detection in high saline solutions relevant to ocean worlds. This study compares two common matrices and three desalting techniques, C4 ZipTip filtration, C18 ZipTip filtration, and on-plate desalting, to improve oligomer detection from saline solutions. We then applied this approach to a unique sample with simulated Enceladus ocean conditions and detected multiple analytes of interest.

## Methods

### Materials

D/L-lactic acid (Sigma-Aldrich, 85% by mass, ACS reagent), l-valine (Sigma-Aldrich, ≥98% reagent grade), l-alanine (Sigma-Aldrich, ≥ 98%), and β-alanine (Fluka, 99%) were used to generate model prebiotic oligomers. Initial oligomers to evaluate desalting were made via two cycles of wetting (85 °C, 6 h, closed vial) and drying (85 °C, 18 h, open vial) with 0.10 M D/L-lactic acid and 0.10 M l-valine. These samples were initially formed in deionized water and were diluted with either acetonitrile or a stock saline solution to final concentrations of 0.15 M Na_2_CO_3_ and 0.075 M NaCl. Saline solution was titrated to pH 7.0 with 1 M HCl.

Deionized water was generated using a Thermo Barnstead system at 18.2 MΩ cm. Acetonitrile (ACN) with 0.1% trifluoroacetic acid (J.T. Baker, LC-MS grade) was obtained from VWR. The MALDI matrices 2,5-dihydroxybenzoic acid (DHB; TCI, 99%) and α-cyano-4-hydroxycinnamic acid (CHCA; Sigma-Aldrich, 99%) were used without additional purification.

### MALDI-TOF Mass Spectrometry

MS analyses were performed on a Bruker autoflex maX MALDI-TOF/TOF in positive ion, reflector TOF mode (*m*/*z* 100–800). Spectra were accumulated such that each had at least 2000 laser shots. MALDI matrices of 20 mg/mL DHB (0.13 M) or saturated CHCA in 50% H_2_O, 50% ACN, and trace TFA were used (1 μL volumes) and were allowed to dry under ambient conditions before samples were added. TOF calibration was performed daily using a custom mixture of amino acids, short peptides, and matrix signals.

### Evaluating Effects of Salts on MALDI Performance

To compare desalting performance, 100 μL of depsipeptide solution, 85 μL of saline stock solution, and 15 μL of 1 M HCl were mixed together. (Acidification is necessary to condition ZipTips, so this was also done for on-target wash samples.) On-plate wash steps used 98% H_2_O, 2% ACN, and trace TFA (1 μL volumes). The wash solution was pipetted onto a dried matrix and analyte and was drawn up and down two times. Wash fluid retained in the pipet tip after mixing was removed as to not introduce any retained salt back into the sample well. After the wash was completed, the samples were allowed to dry again under benchtop conditions until MALDI analysis.

ZipTips usage was consistent with manufacturer instructions. Briefly, tips were conditioned using 99.9% ACN and 0.1% TFA and washed with 98% H_2_O, 2% ACN, and trace TFA before exposure to samples. Samples were drawn up, washed with 98% H_2_O, 2% ACN, and trace TFA, and then eluted using 50% H_2_O, 50% ACN, and trace TFA. Our study utilized 10 μL of C18 tips (bed volume 0.6 μL) and C4 tips (bed volume 0.6 μL). Approximately 1–3 μL of the resulting solution was delivered onto already-dried MALDI matrix and allowed to dry again under room conditions.

### Application: Polymerization of Hydroxy and Amino Acids in Simulated Enceladus Ocean

A solution was formed of 0.10 M lactic acid and 0.10 β-alanine monomers in model Enceladus ocean solution (0.15 M Na_2_CO_3_, 0.075 M NaCl, pH titrated to 9.0). A 200 μL aliquot of this solution was subjected to heating at 85 °C for 1 month (open vial) in a simplified model of chemistry which might take place in a porous, low-water-activity Enceladus core.

## Results and Discussion

An example of salt suppression in MALDI-TOF MS analysis of model prebiotic oligomers is shown in Figure 2. Oligomers consisting of lactic acid (a) and valine (V) made without added salt or pH adjustment were readily detected by MALDI (Figure 2a). However, when the model saline solution was added to the sample, essentially all oligomers of interest disappeared from the spectrum; major signals corresponded to matrix and salt clusters (Figure 2b).

**Figure 2:**
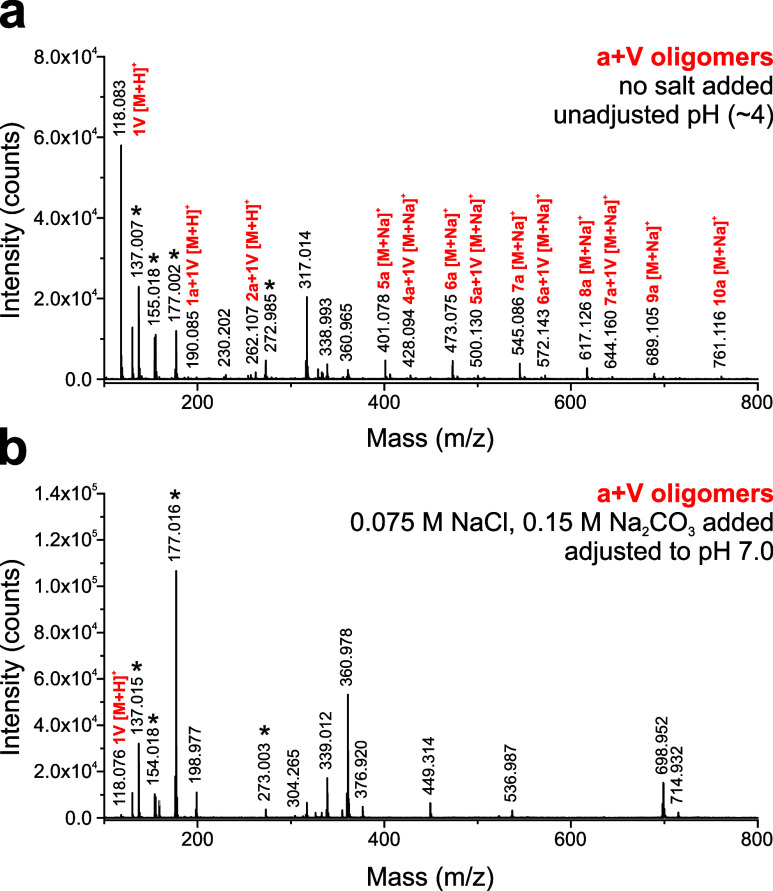
Model ocean world conditions suppress MALDI MS detection of prebiotic oligomers. (a) MALDI mass spectrum of lactic acid and valine (a+V) depsipeptides and oligoesters with negligible salts and no pH adjustment. Oligomers of up to 10 residues in length are detected. (b) MALDI mass spectrum of the same sample mixture when mixed with high saline content at pH 7.0. Due to salt suppression (and possibly some ester hydrolysis), oligomers are not detected (only free valine is observed). In Figure 2b, multiple signals correspond to salt clusters. Note: DHB matrix was used for this sample because the CHCA signal at *m*/*z* 190 overlaps with the 1a+1 V analyte; common DHB matrix signals are marked with asterisks.

As addition of saline solution resulted in signal suppression of the depsipeptides and oligomer analyte signals, we compared the efficacy of two matrices, DHB and CHCA, and three sample preparations, C4 ZipTips, C18 ZipTips, and on-plate washing (Figure 3; full spectra Figures S1–S6), in the presence of model ocean world solution. Zoomed-in mass spectra (*m*/*z* 250–350) should have contained three oligomers of interest: 3a oligoester (theo. [M + Na]^+^ = 257.065 Da), 2a+1 V depsipeptide (theo. [M + Na]^+^ = 284.111 Da), and 4a oligoester (theo. [M + Na]^+^ = 329.086 Da). In this *m*/*z* range, none were detected with C4 or C18 ZipTips and the CHCA matrix (Figure 3a,b). Outside of this range, we did detect monomeric valine as well, but matrix signals dominated (Figures S1–S2). CHCA matrix coupled with on-plate desalting showed reduced overall signal but was able to detect all three of these oligomers with S/*N* > 3:1 (Figure 3c).

**Figure 3:**
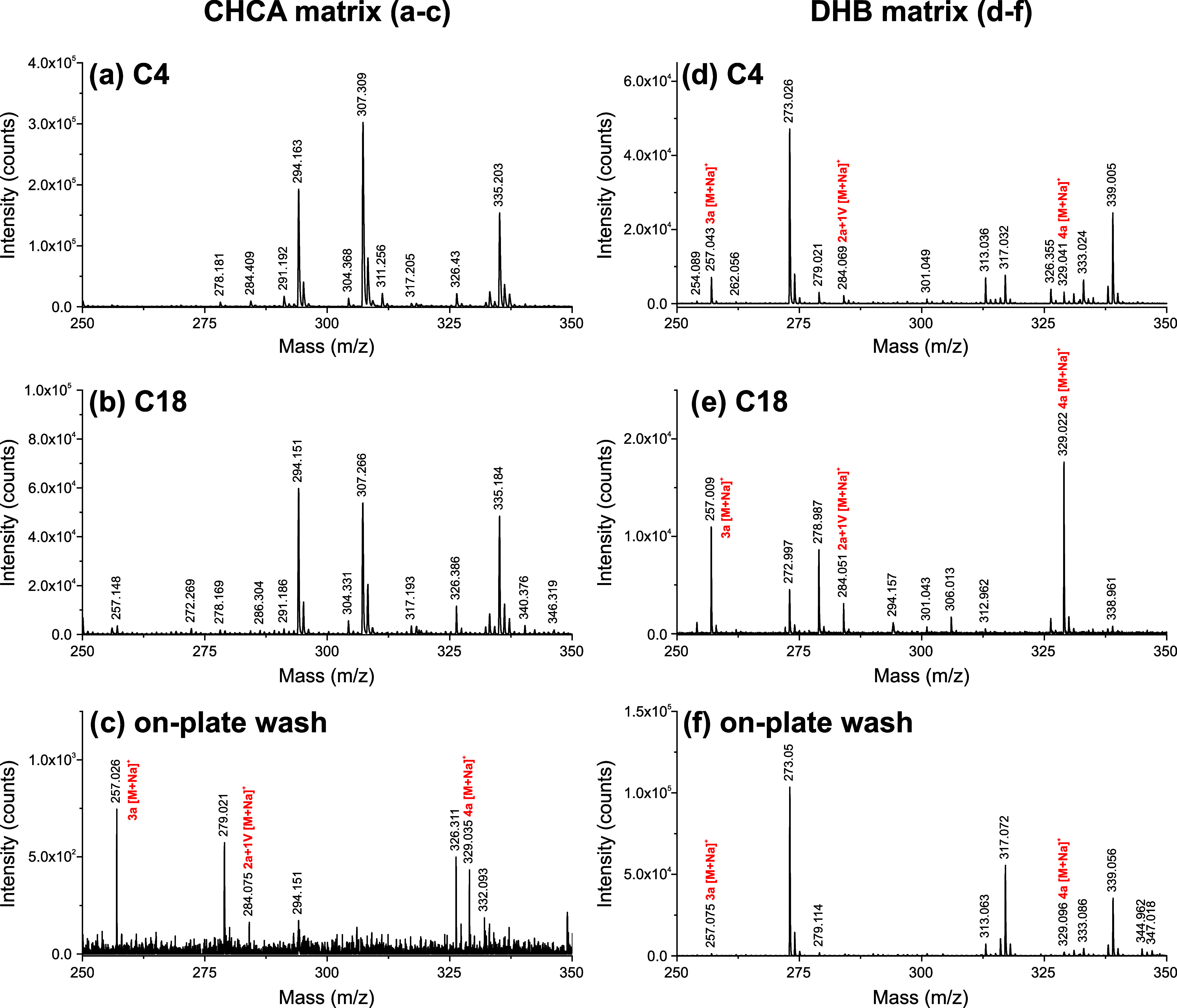
Comparing MALDI-TOF MS performance of C4 ZipTips, C18 ZipTips, and on-target desalting with 98% water, 2% acetonitrile, and trace TFA. Mass spectra are zoomed into *m*/*z* 250–350 for clarity; full spectra are provided in the SI (Figures S1–S6). Analytes of interest in this *m*/*z* range include the 3a oligoester (theo. [M + Na]^+^ = 257.065 Da), 2a+1 V depsipeptide (theo. [M + Na]^+^ = 284.111 Da), and 4a oligoester (theo. [M + Na]^+^ = 329.086 Da). (a–c) Mass spectra of a+V oligomers using the CHCA matrix and three desalting approaches. C4 and C18 ZipTips were not helpful with the CHCA matrix. On-plate desalting reduced overall signal but improved S/N for 3a, 2a+1 V, and 4a oligomers. (d–f) Mass spectra of a+V oligomers using the DHB matrix and three desalting approaches. C18 ZipTips demonstrated optimal performance in terms of analyte detection and S/N.

All three sample preparation techniques tested with DHB produced oligomer signals (Figure 3d–f). DHB paired with C18 ZipTips was the most effective; all three oligomers of interest were detected with significant intensity (Figure 3e). On-plate desalting with DHB (Figure 3f) was able to detect two analytes of interest only. It is possible that on-target washing extracted more analyte from DHB than from CHCA due to differences in their hydrophobicity; however, more study is needed to confirm this hypothesis.

Based on these results, we concluded that the DHB matrix paired with the C18 ZipTip and on-plate desalting paired with the CHCA matrix were both acceptable sample preparation methods. DHB and C18 ZipTip generated the best analyte S/N, but CHCA matrix and on-target washing were the more cost-effective options, as ZipTip consumables are more expensive than standard micropipette tips.

As the goal of this study was to optimize MALDI sample preparation for high saline samples in ocean world laboratory studies, we investigated the polymerization of lactic acid and β-alanine (a noncanonical, prebiotically plausible amino acid) in the presence of a simulated Enceladus ocean. To model low-water-activity environments at Enceladus core-ocean boundary, 0.10 M D/L-lactic acid and 0.10 M β-alanine monomers were placed in 0.075 M NaCl, 0.15 M Na_2_CO_3_, and pH 9 solution and subjected to one month of reaction.

For this sample, we chose CHCA and on-plate washing to mitigate salt interference without the need for ZipTips. As before, we observed a significant reduction in the overall signal intensity with CHCA and on-target washing. We spotted an additional 1 μL of CHCA matrix on top of the washed sample, and the analyte signal for the β-alanine dipeptide increased significantly (Figure 4). We then attempted a series of two and three washes before respotting the CHCA matrix and observed further gains in analyte signal.

**Figure 4:**
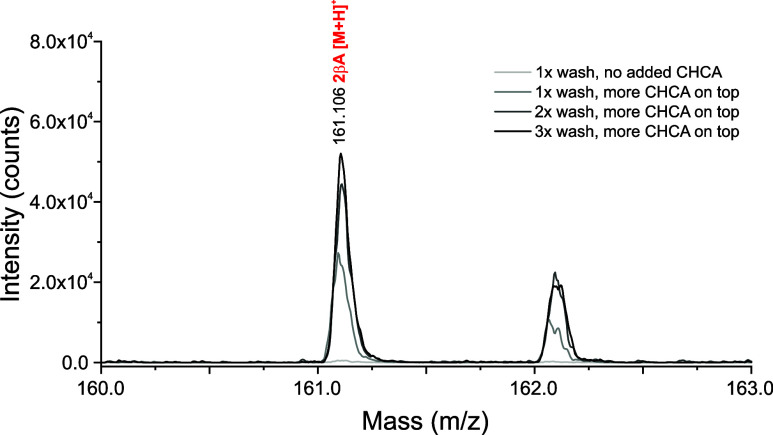
Zoomed-in MALDI-TOF mass spectra of lactic acid (a) + β-alanine (βA) sample after 1 month dry-down in simulated Enceladus ocean (0.075 M NaCl, 0.15 M Na_2_CO_3_, adjusted to pH 9). The βA dipeptide (theo. [M + H]^+^ = 161.093 Da) is shown; signal was improved by repeated washes, followed by additional spotting of the saturated CHCA matrix. The full mass spectrum of 3x wash with matrix readded is provided in the SI (Figure S7).

The resulting spectra showed high matrix signal due to its readdition after washing; however, multiple oligomer signals of interest were detected (Table 2, Figure S7). This wash series was found to mitigate signal suppression from the saline solution, preferentially removing the salts from the solutions and increasing depsipeptide and oligomer signals. Future studies may utilize the CHCA on-target sample preparation to investigate model prebiotic oligomer formation across varying model ocean world conditions.

**Table 2:** Model Prebiotic Oligomers Tentatively Identified from Sample in Figures 4 and S7 Using CHCA Matrix, Three On-Target Washes, and Respotting of CHCA Matrix

oligomer	ion	exp. mass (Da)	theo. mass (Da)	mass error (Da)
2 β-Ala dipeptide	[M + H]^+^	161.106	161.093	+0.013
	[M + Na]^+^	183.107	183.075	+0.032
1 lac +1 β-Ala depsipeptide	[M + Na]^+^	184.090	184.059	+0.031
1 lac +1 β-Ala (cyclic/-H_2_O)	[M + Na]^+^	166.099	166.049	+0.050
1 lac +2 β-Ala depsipeptide	[M + H]^+^	233.133	233.115	+0.018
	[M + Na]^+^	255.118	255.097	+0.021
2 lac +1 β-Ala (cyclic/-H_2_O)	[M + Na]^+^	216.089	216.088	+0.001

Although this study is application-focused, the fundamentals of matrix crystallization warrant a brief discussion. Regarding CHCA and on-target washing, coffee ring formation was pronounced, and crystal homogeneity was poor before washing (Figure S8). While it is likely that some matrix and analyte were removed via washing, it appears that more salt was removed, particularly on surface layers (CHCA was initially spotted beneath the sample). We observed less material clustering and more homogeneity in matrix crystals after washing and matrix respotting (Figure S8). Regarding the use of ZipTips, it is possible that differences in hydrophobicity between CHCA and DHB affected analyte and/or salt incorporation into the matrix and, thus, MS performance between the two.

## Conclusions

With a current NASA mission to Europa and interest in Enceladus also, laboratory studies focusing on prebiotic and/or biological potential within ocean worlds are critical.^19^ This study compared strategies for MALDI-TOF MS of prebiotic chemistry in high saline solutions relevant to ocean worlds. Results indicated that pairing DHB with the C18 ZipTip and pairing CHCA with on-plate desalting are both satisfactory desalting approaches for the detection of relevant analytes. Regarding CHCA, increasing the number of on-plate washes and readding matrix led to higher analyte signal. These strategies can provide a simple, low-volume method for prebiotic analysis in ocean world studies.

## Supplementary Material



## Notes

Full MALDI-TOF mass spectra and camera images of MALDI spots (PDF)
