# Supplementary material for: Sample Preparation for MALDI-TOF Mass Spectrometry of Model Prebiotic Reactions in Simulated Ocean World Environments
Source: ACS Omega. 2025 Oct 20;10(43):51709–15. doi: 10.1021/acsomega.5c07633 (PMC12593968; doi:10.1021/acsomega.5c07633)
Supplement: Supplementary file 1 [file ao5c07633_si_001.pdf]

Supplemental Information

Sample preparation for MALDI-TOF mass spectrometry of model prebiotic reactions in simulated ocean world environments

Katherine A. Dzurilla,[1] Elin C. Herndon,[2] Laura M. Barge,[1] and Jay G. Forsythe[2]*

(1) NASA Jet Propulsion Laboratory, California Institute of Technology, Pasadena, CA 91109
(2) Department of Chemistry and Biochemistry, College of Charleston, Charleston, SC 29424

*corresponding author

Jay G. Forsythe, Ph.D.
Department of Chemistry and Biochemistry
College of Charleston
66 George Street
Charleston, SC 29424-0001
USA

Email: forsythejg@cofc.edu
Phone: (+1) 843-953-5052

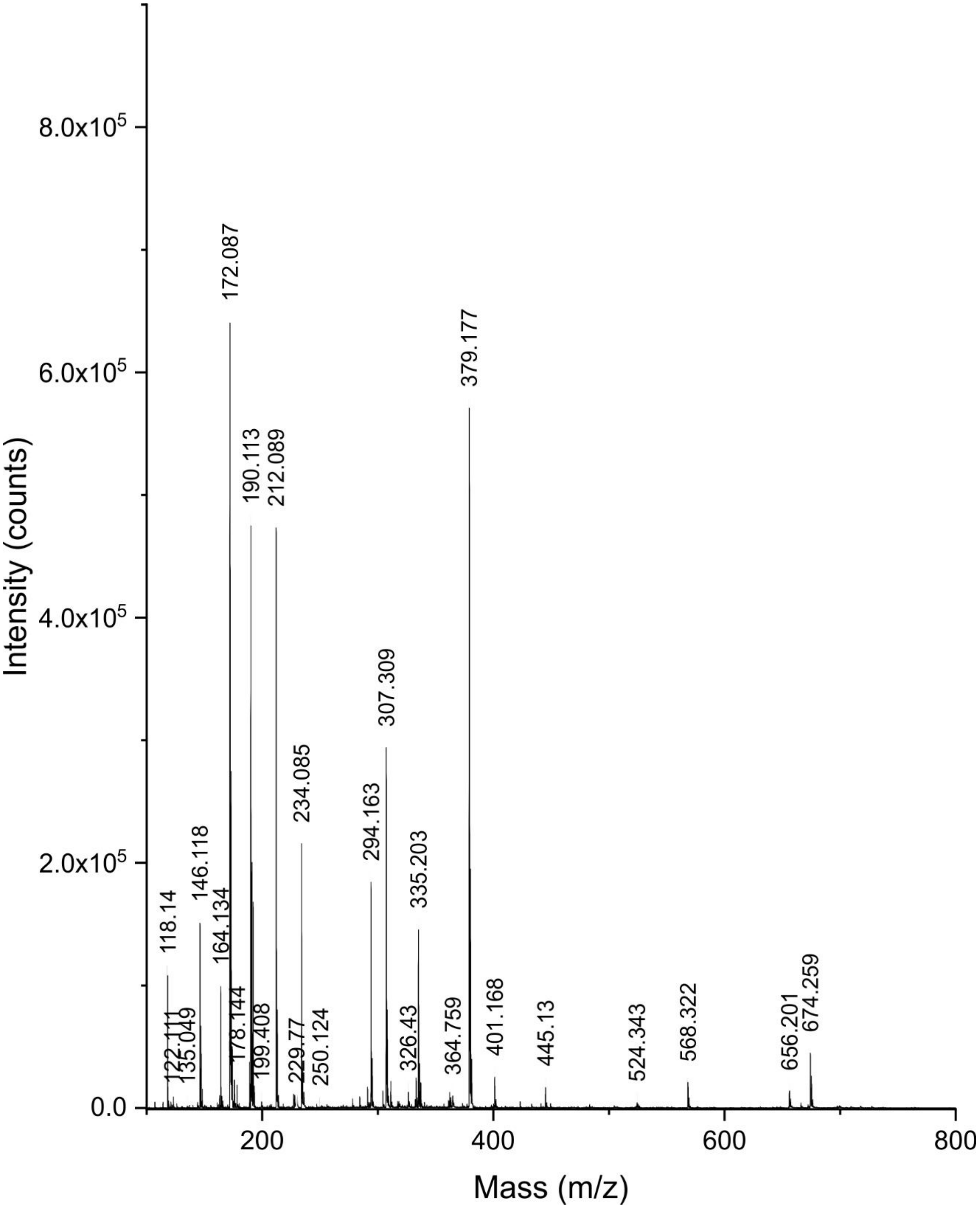

Figure S1. Full MALDI-TOF mass spectrum of lactic acid + valine reaction products from **Fig. 3a** in main text (CHCA matrix, C4 ZipTips).

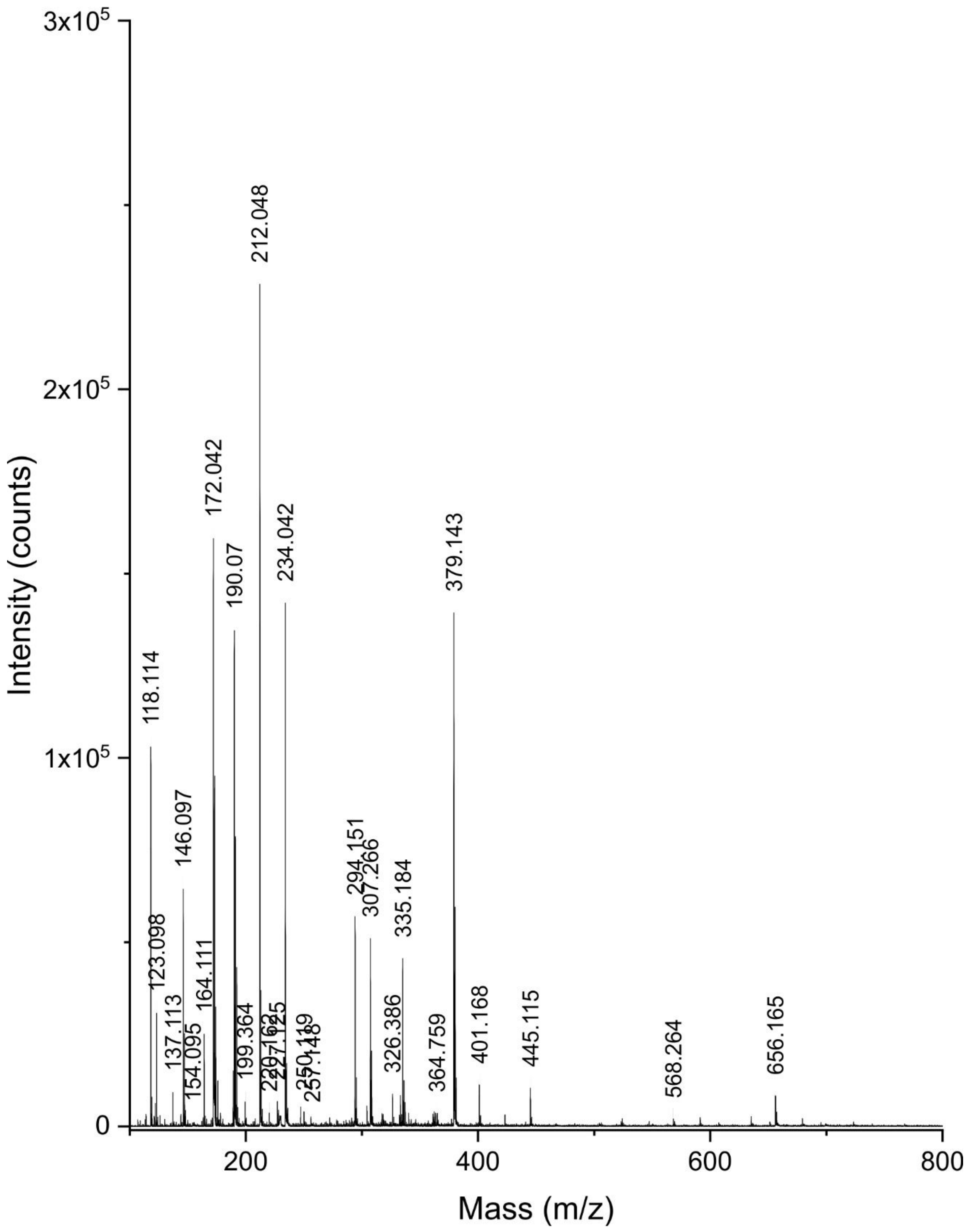

Figure S2. Full MALDI-TOF mass spectrum of lactic acid + valine reaction products from **Fig. 3b** in main text (CHCA matrix, C18 ZipTips).

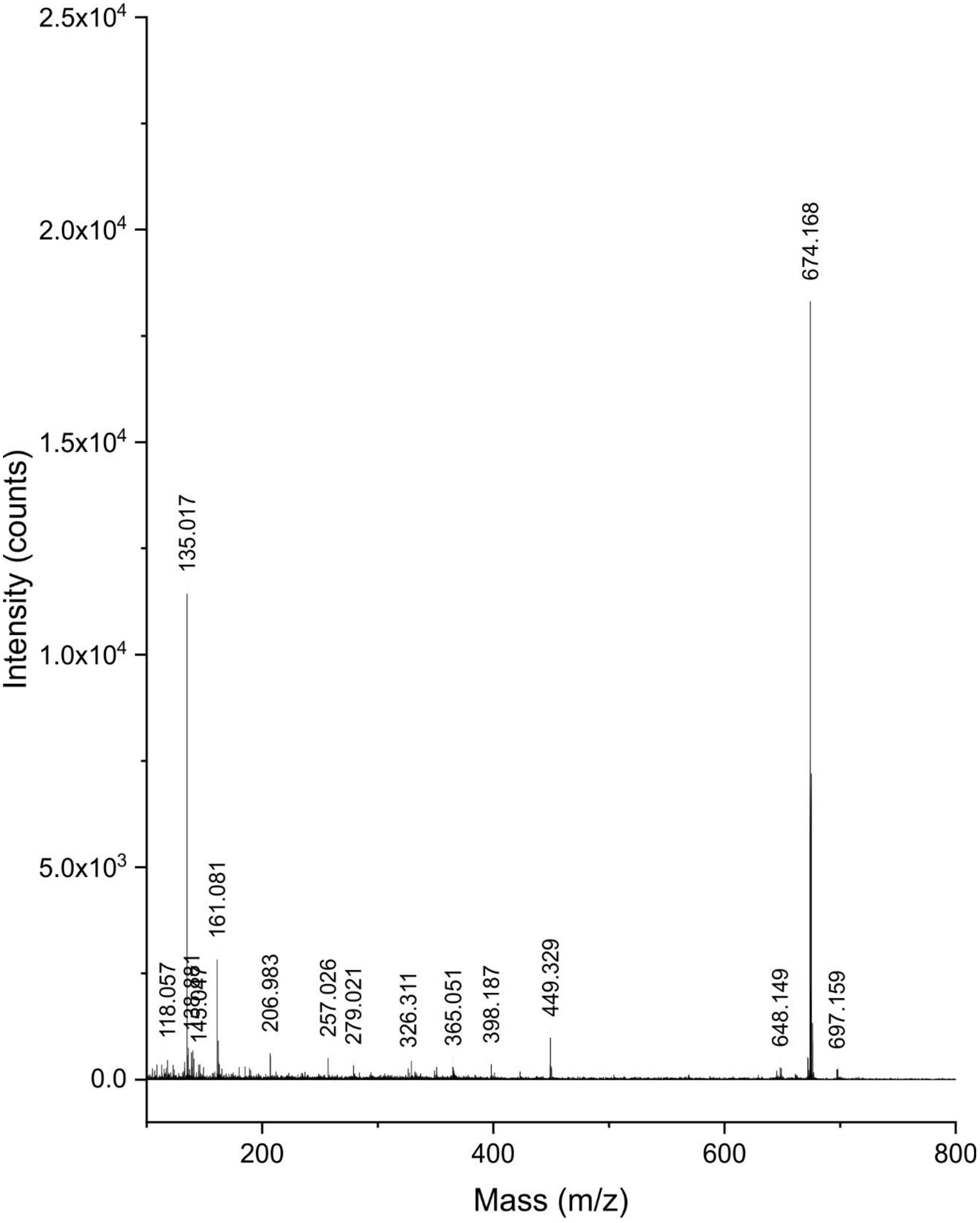

Figure S3. Full MALDI-TOF mass spectrum of lactic acid + valine reaction products from **Fig. 3c** in main text (CHCA matrix, one on-plate wash, no matrix re-added).

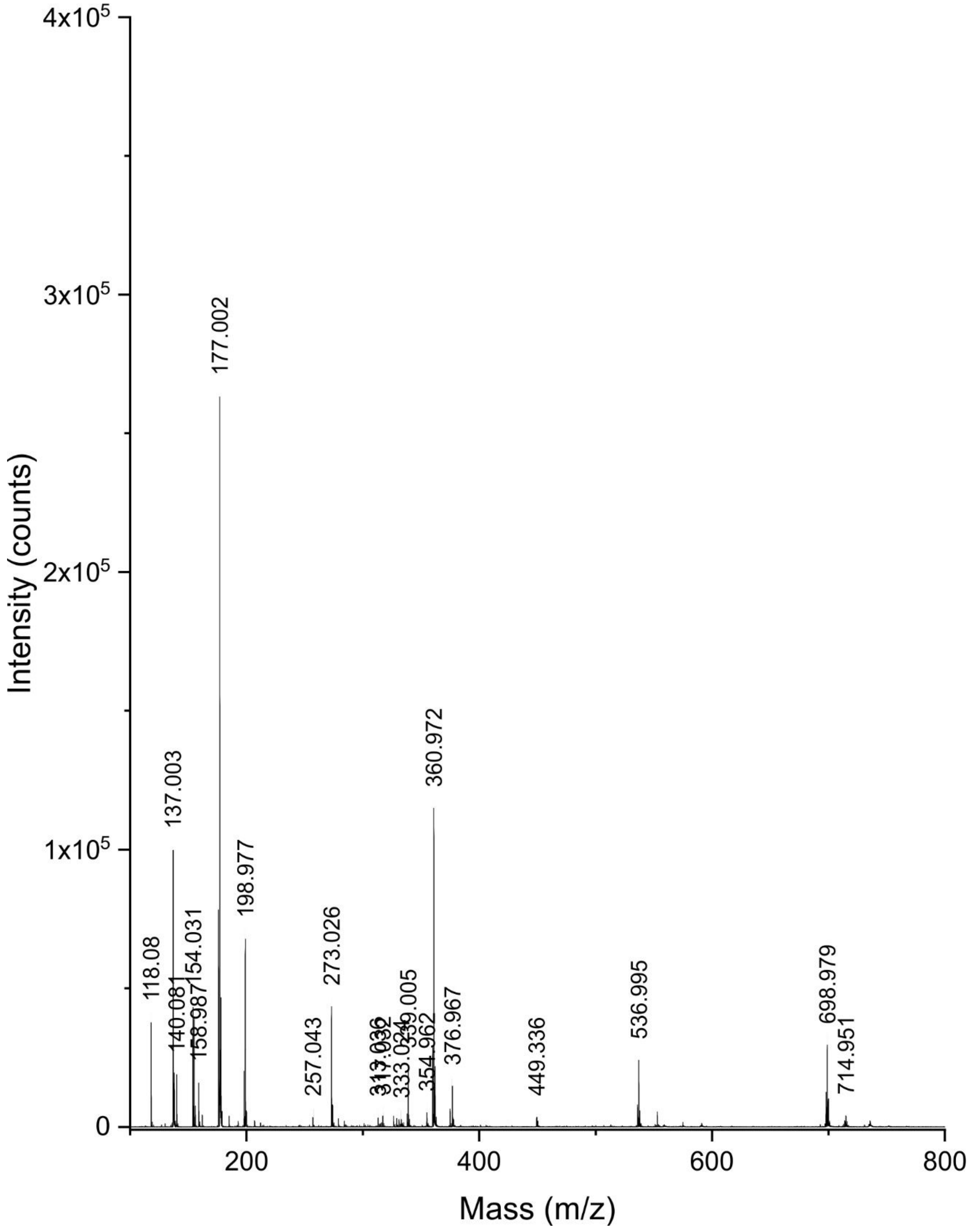

Figure S4. Full MALDI-TOF mass spectrum of lactic acid + valine reaction products from **Fig. 3d** in main text (DHB matrix, C4 ZipTips).

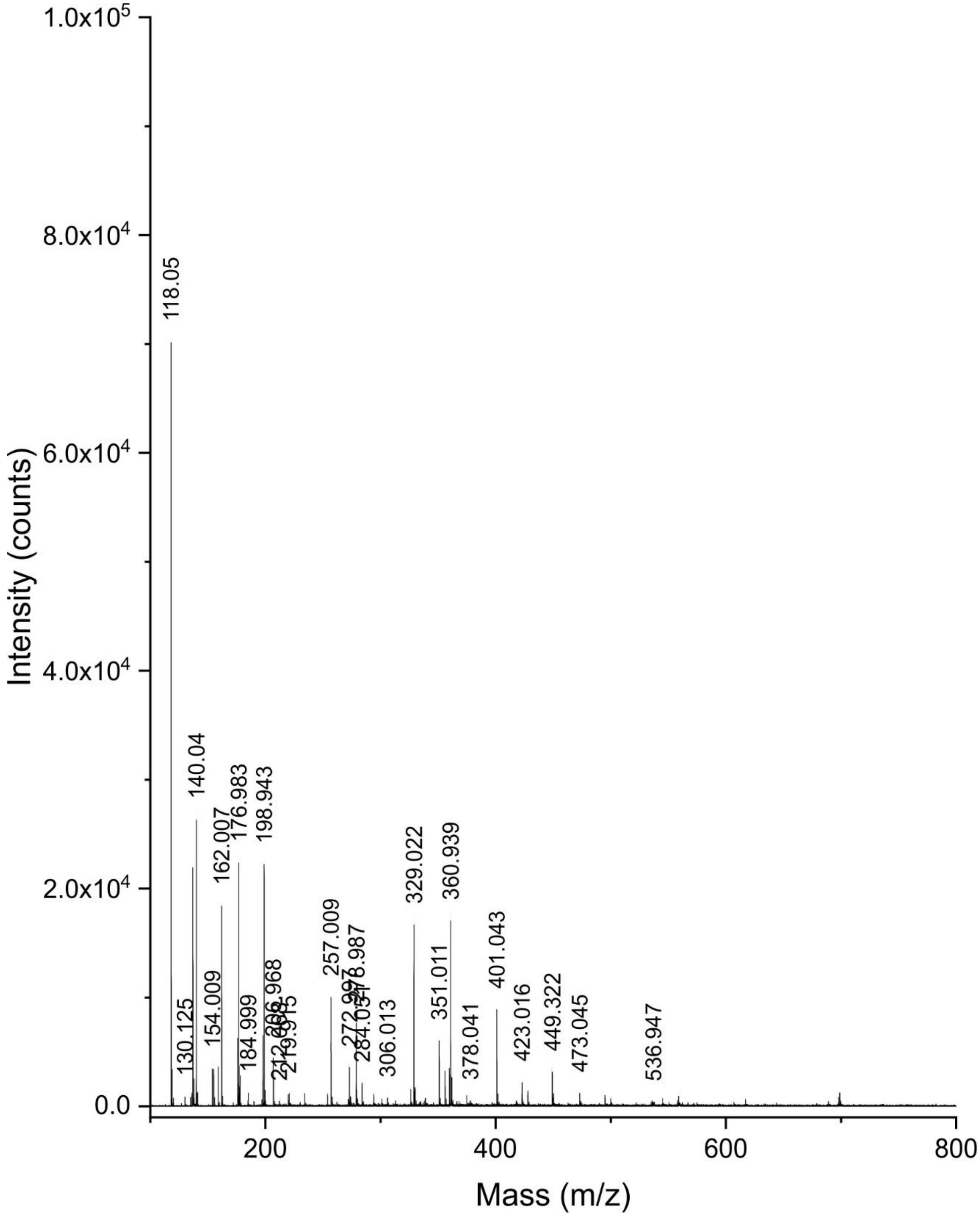

Figure S5. Full MALDI-TOF mass spectrum of lactic acid + valine reaction products from **Fig. 3e** in main text (DHB matrix, C18 ZipTips).

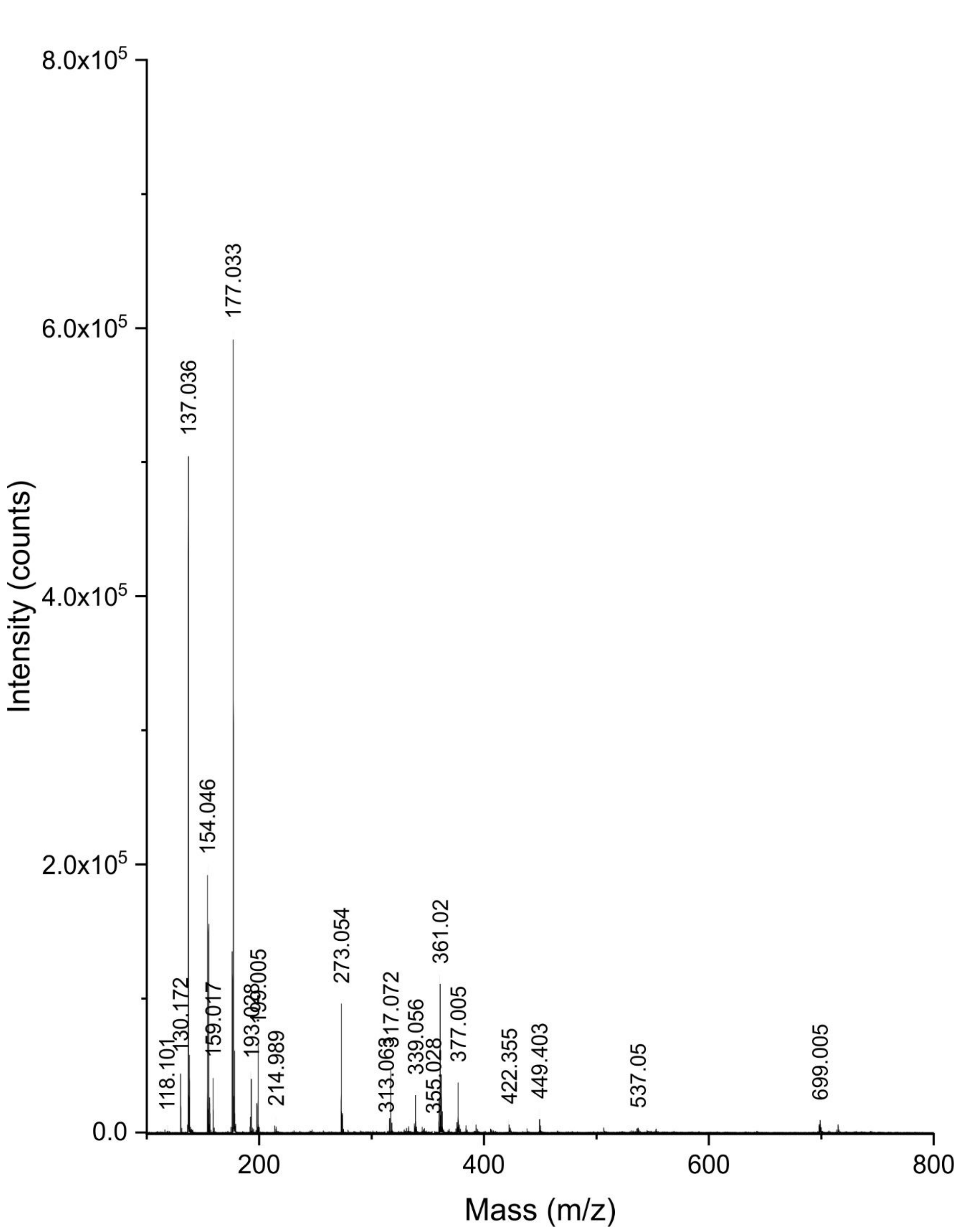

Figure S6. Full MALDI-TOF mass spectrum of lactic acid + valine reaction products from **Fig. 3f** in main text (DHB matrix, one on-plate wash, no matrix re-added).

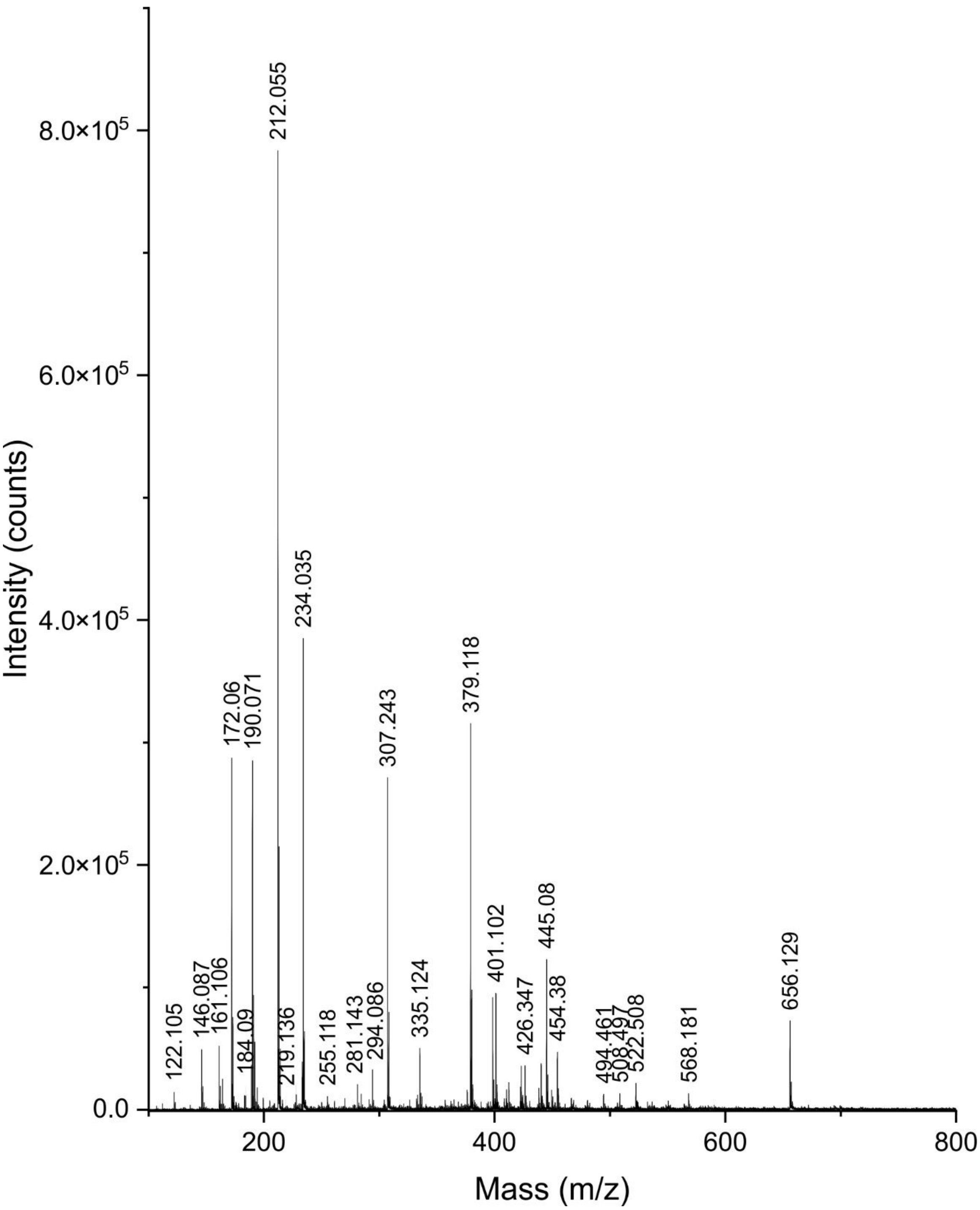

Figure S7. Full MALDI-TOF mass spectrum of lactic acid + beta-alanine reaction products from **Fig. 4** in main text (1 month reaction at 85°C in 0.15 M Na_2CO_3, 0.075 NaCl, adjusted to pH 9.0; CHCA matrix, three on-plate washes followed by re-addition of CHCA matrix).

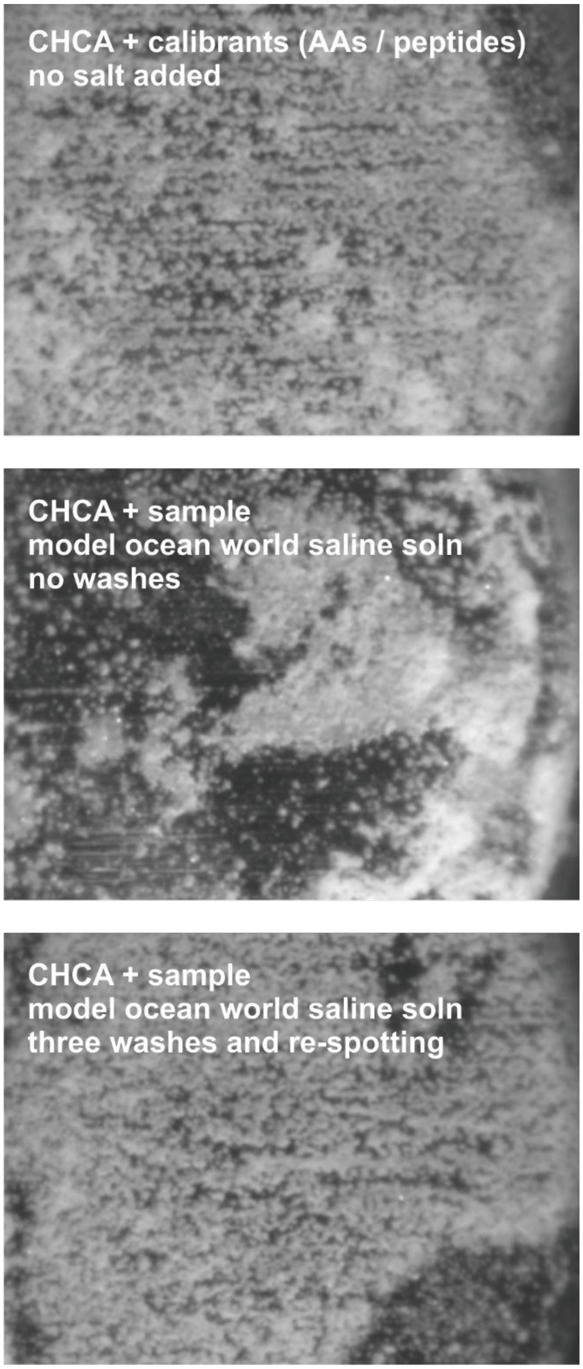

Figure S8. Captured images of crystallization and heterogeneity on the MALDI stainless steel target. As described in the main text, CHCA matrix was spotted first and allowed to dry. In the top panel, calibrants and no added salt were spotted on top. In the middle panel, lactic acid and beta-alanine sample containing model ocean world solution was spotted. Coffee ring effects and material clustering were observed. After three washes and re-spotting of CHCA matrix, it looked similar to the original matrix and calibrant target spot.
